# Limitations of lymphoblastoid cell lines for establishing genetic reference datasets in the immunoglobulin loci

**DOI:** 10.1371/journal.pone.0261374

**Published:** 2021-12-13

**Authors:** Oscar L. Rodriguez, Andrew J. Sharp, Corey T. Watson

**Affiliations:** 1 Department of Biochemistry and Molecular Genetics, University of Louisville School of Medicine, Louisville, KY, United States of America; 2 Department of Genetics and Genomic Sciences, Icahn School of Medicine at Mount Sinai, New York, NY, United States of America; University of California San Francisco, UNITED STATES

## Abstract

Lymphoblastoid cell lines (LCLs) have been critical to establishing genetic resources for biomedical science. They have been used extensively to study human genetic diversity, genome function, and inform the development of tools and methodologies for augmenting disease genetics research. While the validity of variant callsets from LCLs has been demonstrated for most of the genome, previous work has shown that DNA extracted from LCLs is modified by V(D)J recombination within the immunoglobulin (IG) loci, regions that harbor antibody genes critical to immune system function. However, the impacts of V(D)J on short read sequencing data generated from LCLs has not been extensively investigated. In this study, we used LCL-derived short read sequencing data from the 1000 Genomes Project (n = 2,504) to identify signatures of V(D)J recombination. Our analyses revealed sample-level impacts of V(D)J recombination that varied depending on the degree of inferred monoclonality. We showed that V(D)J associated somatic deletions impacted genotyping accuracy, leading to adulterated population-level estimates of allele frequency and linkage disequilibrium. These findings illuminate limitations of using LCLs and short read data for building genetic resources in the IG loci, with implications for interpreting previous disease association studies in these regions.

## Introduction

Lymphoblastoid cell lines (LCL) are generated by infecting B cells with the Epstein Barr Virus (EBV) [1] to create immortalized cell lines. Various consortia, including The International HapMap Project [2, 3], 1000 Human Genome Project (1KGP) [4–6], Genome In A Bottle [7, 8] and Human Genome Structural Variation Consortium [9] have used DNA from LCLs to characterize common genetic variation, generate gold standard sets of small insertions and deletions (indels), and comprehensively genotype structural variants (SV). Variant call sets from these initiatives have been instrumental to the genomics community, and are routinely used in genome-wide association studies (GWAS) and other genetic studies. Genome-wide genotypes from LCLs have been shown to be nearly identical to genotypes derived from whole blood or peripheral blood mononuclear cells (PBMC) using SNP arrays [10], whole exome sequencing [11, 12] and short read whole genome sequencing [13]. However, somatic LCL-associated alterations are present in particular regions of the genome, namely within the immunoglobulin (IG) heavy (IGH) and light (lambda, IGL; kappa, IGK) chain loci. These alterations could impact sequencing, mapping, and genotype results in these regions, with potential implications for downstream uses of these data.

**Fig 1:**
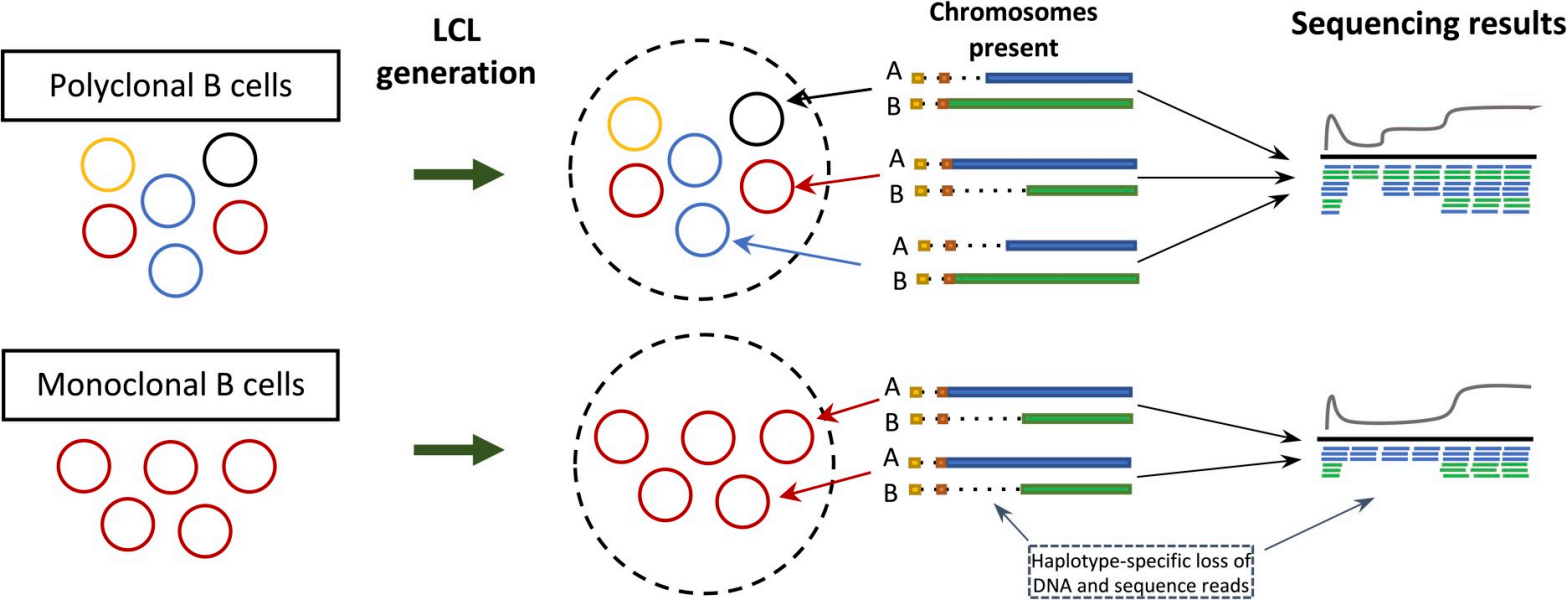
Generation of LCLs can lead to loss of sequencing data. LCLs are generated from a pool of B cells. A pool of polyclonal B cells will contain different V(D)J recombination events, mitigating the impacts of V(D)J associated deletions on generated sequencing data. In such cases, we would expect read coverage from both chromosomes across the IGH locus. In contrast, LCLs representing monoclonal B cells (*i*.*e*. B cells in which the same V, D, and J genes are selected by V(D)J recombination), would result in the loss of haplotype-specific sequencing data.

The IG loci encode the variable (V), diversity (D), joining (J) and constant (C) gene segments that serve as the building blocks for the expression of functional B cell receptors (BCRs) and antibodies (Abs). During B cell development, the V, D, and J gene segments within each IG locus (V and J in the case of IGL and IGK) are somatically rearranged through a process called V(D)J recombination [14]. During this process, intervening DNA between recombined V, D, and J segments is excised. The size of these somatic deletions on the recombined chromosome depends on the selected V, D, and J gene segments, but can extend 100’s of Kb, and will vary from cell to cell. Collectively, DNA isolated across a pool of B cells (e.g., naive B cells) representing many independent V(D)J recombination events would be expected to represent each germline haplotype present in a given sample (Fig 1). In contrast, a pool of B cells originating from a single or dominant expanded B cell would harbor DNA not fully representative of both paternal and maternal germline haplotypes within the IG loci (Fig 1). In the latter instance, genotyping methods dependent on different read alignment signatures such as read depth/coverage, discordant read mapping, soft-clipped or split reads could produce inaccurate germline genotypes.

Recent long read sequencing and assembly of complete IGH haplotypes from selected 1KGP individuals has revealed the presence of V(D)J recombination associated deletions [15], indicating that genotypes derived from such samples within regions impacted by V(D)J recombination are inaccurate. Critically, the use of long read sequencing facilitates the direct detection of deletions induced by V(D)J recombination within LCLs, allowing for such alterations to be accounted for in data analysis and interpretation. While it has been speculated previously that V(D)J recombination would have negative impacts on LCL-derived short read sequencing data [16–18], this has not been comprehensively investigated. Given this, we sought to evaluate the extent of sample-level V(D)J recombination in LCL-derived short read sequencing data from the 1KGP, and assess downstream impacts of these somatic events. We demonstrate that short read data is affected by V(D)J recombination and, depending on the sample, is derived from either single dominant or multiple B cell clones. We show that variation in sample clonality is associated with variability in genotyping accuracy, negatively impacting estimates of allele frequency and linkage disequilibrium (LD). These data raise important considerations for using 1KGP short read derived genotypes to augment genetic association studies in the IG loci, and in addition to other issues discussed previously [16–18], may further explain the paucity of disease associations within these complex regions of the genome.

## Materials/Subjects and methods

### 1000 human genome project data

Paired-end 150 bp PCR-free 30X coverage Illumina data on 2504 individuals from the 1KGP [19] was downloaded from the European Bioinformatics Institute (EBI) under the study ID ERP114329. 1KGP phase 3 [4] SNPs were downloaded from ftp://ftp.1000genomes.ebi.ac.uk/vol1/ftp/data_collections/1000_genomes_project/release/20190312_biallelic_SNV_and_INDEL/ALL.chr14.shapeit2_integrated_snvindels_v2a_27022019.GRCh38.phased.vcf.gz.

### Assessment of insert sizes, read depth, and the identification of V(D)J recombination events

Insert sizes for read pairs within each sample were calculated using the Picard (https://github.com/broadinstitute/picard) CollectInsertSizeMetrics tool with the following parameters: `—DEVIATIONS 1000000—MINIMUM_PCT 0`for reads spanning chr14:105862198–107043718. The same tool and parameters were used for ten random 1.2 MB windows across the genome. To calculate read depth across the IGHD and V regions, we used samtools [20] (IGHD, hg38, chr14:105,865,458–105,939,756; IGHV, hg38, chr14:105,939,756–106,883,718). To identify read pairs within each sample representing V(D)J recombination events, we utilized a custom python script. For each sample, the number of clones was calculated by counting the number of unique IGHV and IGHJ gene segment pairs detected. The frequency of each “clone” was calculated by determining the number of reads mapping to a unique IGHV and IGHJ gene segment pair, and taking this as a fraction of the total number of reads assigned to any IGHV/IGHJ pair. The sizes of somatic deletions were determined by calculating the genomic distances between the IGHJ and IGHV gene segments utilized in a given V(D)J event.

### Analysis of heterozygosity, allele frequency, and LD

The percentage of heterozygous SNPs was calculated for each sample in the centromeric and telomeric region of the selected V gene segment in the dominant clone. Two VCFs were created for each region using tabix to select the region of interest on the 1KGP phase 3 VCF. The telomeric region was set to start at the 3’ end of *IGHV6-1* (chr14:105,939,756; GRCh38). This represents the beginning of the IGHV gene segment region within the IGH locus, which extends from this position to the telomere of chromosome 14. The fraction of heterozygous SNPs was calculated by counting the number of heterozygous SNPs over the total number of SNPs in each VCF.

To assess the effects of V(D)J recombination on allele frequency estimates and LD, we subsetted the 1KGP phase 3 genotype call set to samples from the “AFR” superpopulation, further selecting samples representing two extremes of clonality (0–25%, n = 38; 75–100%, n = 38). Allele frequencies for each set of samples were calculated using the vcftools `freq`tool. The LD scores were calculated for the African samples of each set of samples using the vcftools `hap-r2`tool with the parameters `—ld-window 1000000—min-r2 0.01`.

## Results

### Detecting signatures of V(D)J recombination

**Fig 2:**
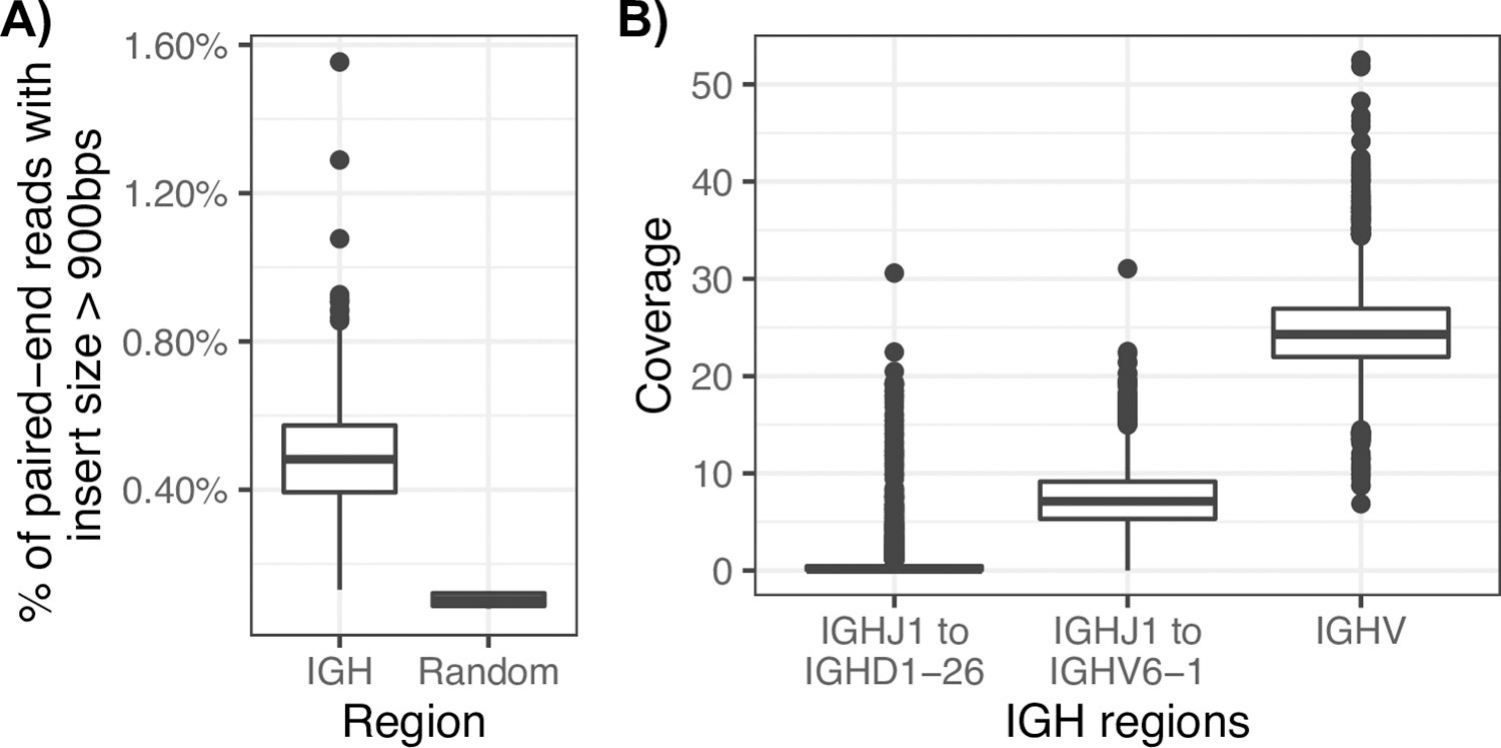
Signatures of V(D)J recombination in paired-end WGS data. (A) Percentage of paired-end read pairs with insert sizes greater than 900 bps in IGH and random genome-wide 1 Mb windows. (B) WGS coverage (1) between *IGHJ1*, the IGHJ gene closest to the telomeric end, and *IGHD1-26*, the second closest gene to the IGHJ gene cluster; (2) between *IGHJ1* and *IGHV6-1*, the IGHV gene closest to the IGHD and IGHJ gene cluster; and (3) the entire IGHV region.

To determine the effect of V(D)J recombination on short read whole genome sequencing (WGS) data in IGH, we used paired-end 150 bp PCR-free Illumina data on 2,504 individuals from the 1KGP, recently resequenced to high coverage [19]. The occurrence of V(D)J recombination results in large somatic deletions within the IGH locus spanning the IGHJ, IGHD, and IGHV regions. To assess signatures of these somatic deletions we first analyzed paired-end mapping distances, as measured by the predicted “insert sizes”. We reasoned that the presence of V(D)J recombination events would result in larger insert sizes, and that these would be enriched within IGH. To assess this, we calculated the number of read pairs with an insert size >900 bp (two times the library DNA insert size) at 10 random 1.2 MB windows (the length of the IGH locus in GRCh38) across the genome from five individuals chosen at random. Across these regions in the selected individuals, we observed that 0.08% to 0.12% (mean = 0.10%) of the paired-end reads contained an insert size greater than 900 bps. In contrast, across all samples, 0.13% to 1.55% (mean = 0.49%) of paired-end reads in IGH contained an insert size greater than 900 bps. This is almost a 5-fold increase in the number of paired-end reads with a larger insert size (Fig 2A).

To further evaluate the effect of V(D)J recombination, we calculated the coverage over the IGHD region. During B cell development, through the formation of the pre-B cell receptor, V(D)J recombination results in a loss of DNA between selected IGHJ and IGHD gene segments on each homologous chromosome [21]. Therefore, if V(D)J recombination has occurred, there should be limited to no coverage within the IGHD region. The closest IGHJ and IGHD gene segment pair, *IGHJ1* and *IGHD7-27*, are within 100 bps, so we assessed coverage between *IGHJ1* and *IGHD1-26* (the second closest IGHD gene and roughly ~15 Kb away from IGHJ1) across all 2504 individuals, and observed a mean coverage of 0.76X (range = 0–30.6). We also observed a mean coverage of 7.30X between *IGHJ1* and *IGHV6-1*, in contrast to a mean coverage of 24.68X across the entirety of the IGHV region (Fig 2B).

We next used the paired-end data to directly detect V(D)J recombination events by identifying read pairs with one mate overlapping an IGHJ gene segment and the other mate overlapping an IGHV gene segment. We detected read pairs representing V(D)J recombinants in all 2,504 samples, with a mean of 22 V(D)J associated read pairs per sample (range = 2–66). V(D)J rearrangements utilizing *IGHJ4* and *IGHV3-23* were the most common (S1 Fig). Taken together, these three pieces of evidence, increased insert sizes, decreased coverage over IGHD, and the direct detection of read pairs overlapping V(D)J recombination events, indicate that in fact LCLs utilized by the 1KGP cohort have undergone V(D)J recombination.

### Sequencing data derived from multiple B cell clones

**Fig 3:**
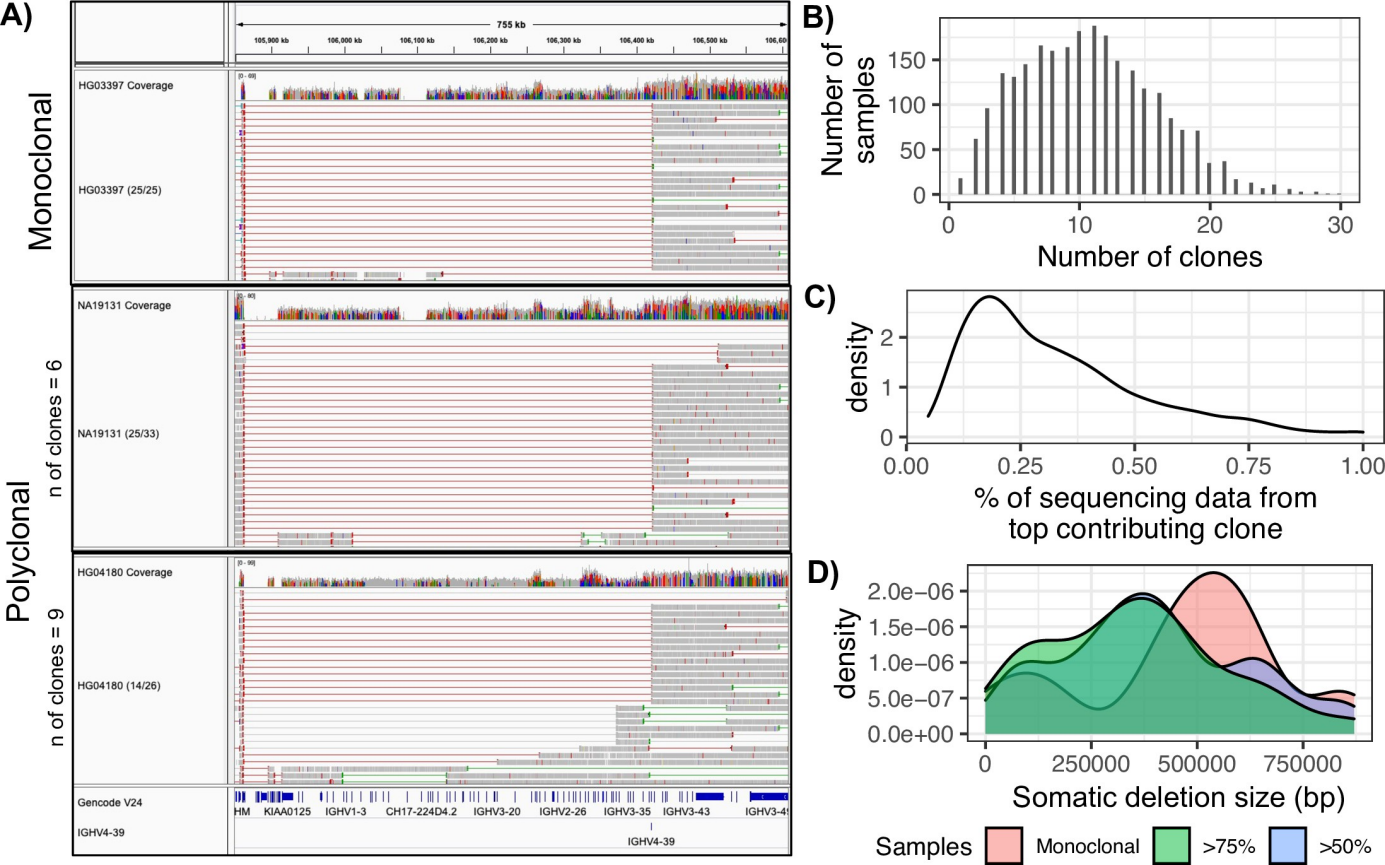
Data signatures of clonality in different samples. (A) IGV screenshots showing three representative samples with different degrees of clonality, all of which are represented by a dominant clone involving the V(D)J selection of *IGHV4-39*. Red and gray lines represent long paired read insert lengths. HG03397 has 25 read pairs aligning to *IGHV4-39* and are labelled as monoclonal. NA19131 and HG04180, which are defined as polyclonal, have 25/33 and 14/26 read pairs aligning to *IGHV4-39*. (B) The numbers of samples represented by varying numbers of identified “clones” (unique IGHJ-IGHV pairs). (C) Density plot showing the percentage of sequencing data derived from the dominant clone across all samples. (D) Density plot showing the sizes of somatic deletions associated with the dominant clone across all samples, grouped by whether they are monoclonal or polyclonal; polyclonal samples, specifically, are partitioned into those in which either >50% or >75% of sequencing data was represented by the dominant clone.

Given that V(D)J recombination has occurred across the cohort, we sought to determine whether all samples were affected equally. We reasoned that samples with sequencing data from a single B cell clone (monoclonal) or from multiple clones (polyclonal) will be differentially impacted by the effects of V(D)J (Fig 3A). We therefore sought to determine the number and frequency of V(D)Js in each sample. To do this, we assigned read pairs overlapping V(D)J events to their respective combination of IGHJ and IGHV gene segments. Reads across a given dataset harboring the same IGHJ/IGHV combination were grouped, and used as a proxy for a group of clonally related sequences. We thus took the number of grouped sequence reads to represent the frequency of a particular IGHJ/IGHV combination, which we heretofore refer to as a clone (Fig 3A). Following this, we calculated the number of unique IGHJ/IGHV combinations (“clones”) present in each sample, and their relative frequency, allowing us to approximate the number of different B cell clones represented in a sample. We found that sequences across samples in the cohort were derived from a mean of 10.76 B cell clones (range = 1–30; Fig 3B). From this, 18 samples were predicted to be monoclonal, represented by reads mapping to only a single clone. We also reasoned that polyclonal samples, represented by many clones, but in which the majority of sequencing data is predicted to be derived from a dominant clone will have profiles similar to those observed for monoclonal samples. To estimate this, we asked what proportion of all sequences containing a V(D)J recombination event were represented by the most frequently observed clone. In doing this, we found that in 407 and 88 samples, respectively, 50% and 75% of all reads containing V(D)J recombination events mapped to a single clone. This indicated that although these samples had a polyclonal signature, the majority of sequencing data was likely derived from a single dominant clone. Based on this approximation, across samples, the top clone identified contributed on average 32.94% (range = 5–100%) of the sequencing data (Fig 3C). We observed a modest population bias, with African and European individuals containing a slightly greater fraction of sequencing data from a single clone (S2 Fig).

Depending on the clonality of the sample and the gene segments involved in the primary V(D)J recombination event present within a sample, the size of the region impacted was expected to vary. We estimated this in each sample based on the most prevalent IGHJ/IGHV gene segment combination observed (*i*.*e*., the dominant clone), revealing that in many samples the predicted size of these somatic deletions was extensive. In the 18 monoclonal samples, we found that an average of 464 Kb of the IGH locus was impacted by V(D)J recombination (range = 74.3–937.2 Kb). In samples that were polyclonal, but still represented by a dominant clone (*i*.*e*., those in which >50% and >75% of sequence data were derived from a single IGHJ/IGHV combination), the regions impacted by V(D)J recombination were found to be on average 356 Kb (50%, range = 74.3–945.0 Kb) and 401 Kb (75%, range = 74.3–945.0 Kb) in size (Fig 3D). Regions affected by V(D)J recombination in each sample are provided in S1 Table.

### The effects of V(D)J recombination on genotype call sets

We reasoned that V(D)J events could impact the accuracy of sample- and population-level genotypes in two primary ways: 1) the loss of DNA and reduced read coverage over extended regions of the locus would result in the increased likelihood of calling homozygous genotypes at heterozygous positions; 2) somatic hypermutations (SHMs) in recombined gene segments could introduce false heterozygous SNPs. Furthermore, these effects would likely be more prominent in monoclonal samples, as well as polyclonal samples represented by dominant clones.

**Fig 4:**
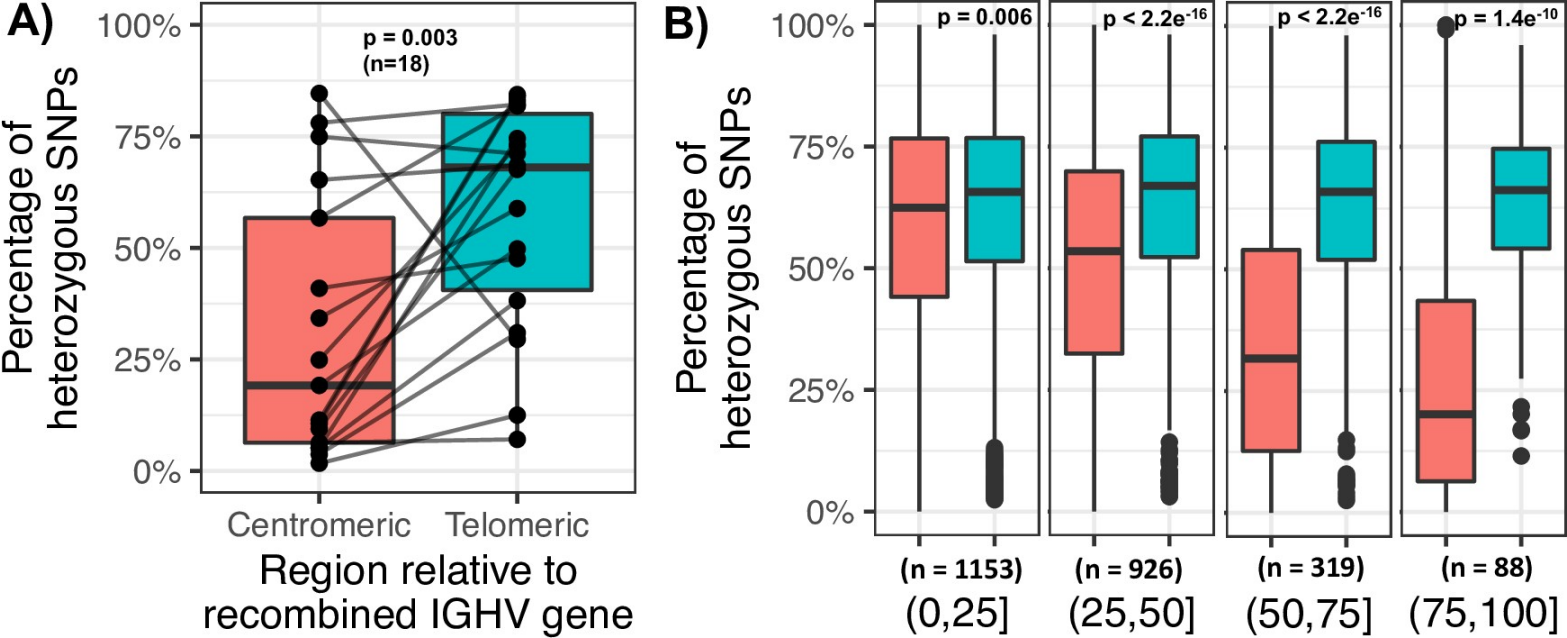
Differences in heterozygosity in regions centromeric and telomeric of IGHV gene segments selected during V(D)J recombination, and the relationship with clonality bias. (A) A boxplot showing within sample differences in the percentage of heterozygous SNPs in the regions centromeric and telomeric of the IGHV gene selected for V(D)J recombination across monoclonal samples (n = 18). (B) Similar to (A), boxplots showing within sample differences in the percentage of heterozygous SNPs in the regions centromeric and telomeric of the IGHV gene selected for V(D)J recombination across all samples, partitioned by the degree of clonality.

To investigate these potential impacts, we first evaluated the number of heterozygous SNPs in the centromeric and telomeric regions of the recombined IGHV gene segment in monoclonal samples. To do this, positions were partitioned into those residing centromeric of the 3’ most base of the recombined IGHV gene segment, and those residing telomeric of this base, inclusive of the IGHV gene segment sequence. We reasoned that if heterozygous positions were erroneously identified as homozygous due to V(D)J recombination, we would expect to observe more homozygous genotypes centromeric of the recombined IGHV segment. Indeed, we found that the mean percentage of heterozygous variants telomeric of the IGHV gene segment used for V(D)J recombination was 3.4 fold higher than the mean percentage of heterozygous variants centromeric of the IGHV gene segment (*P* = 0.003, two-sided paired Wilcoxon test; Fig 4A). To assess this effect in polyclonal samples, we split individuals into four groups representing varying degrees of clonal bias, based on whether 0–25%, 25%-50%, 50%-75% and 75%-100% of sequencing data within a given sample came from the dominant clone. In samples with 0 to 25% of sequencing data from the dominant clone, for which we expected to observe minimal impacts on genotyping, the mean percentage of heterozygous variants telomeric (62%) of the selected recombined IGHV gene segment was 1.04-fold higher than in the centromeric region (59%; *P* = 0.006, two-sided paired Wilcoxon test; Fig 4B). We noted significant differences in the remaining three groups as well (Fig 4B), but the average fold-differences between heterozygous percentages telomeric to the V(D)J event relative to centromeric to the V(D)J event were greater, and as expected, was greatest in samples from the 75%-100% group (2.08-fold).

Additionally, we evaluated the number of heterozygous SNPs overlapping IGHV gene segments most frequently selected for V(D)J recombination in each sample. When stimulated by an antigen, B cells acquire SHMs within IG V, D, and J gene segments as a means to increase antibody affinity [22]. Therefore, SHMs in the recombined IGHV gene segments are more likely to be detected in monoclonal or polyclonal samples with reads primarily derived from a dominant clone. Indeed, there was a significant positive correlation (R = 0.33, p-value < 2.2e-16) between the contribution of sequencing data from the dominant clone and the number of the heterozygous variants within the IGHV gene segment selected by V(D)J recombination (S3A Fig). We also directly compared the number of heterozygous genotypes within the recombined IGHV gene segments to non-recombined IGHV gene segments across all samples, and observed an average of 1.92 heterozygous positions in the recombined IGHV gene segments, compared to 0.5 in the non-recombined IGHV gene segments (S3B Fig).

### The effects of V(D)J recombination on estimates of allele frequency and linkage disequilibrium

**Fig 5:**
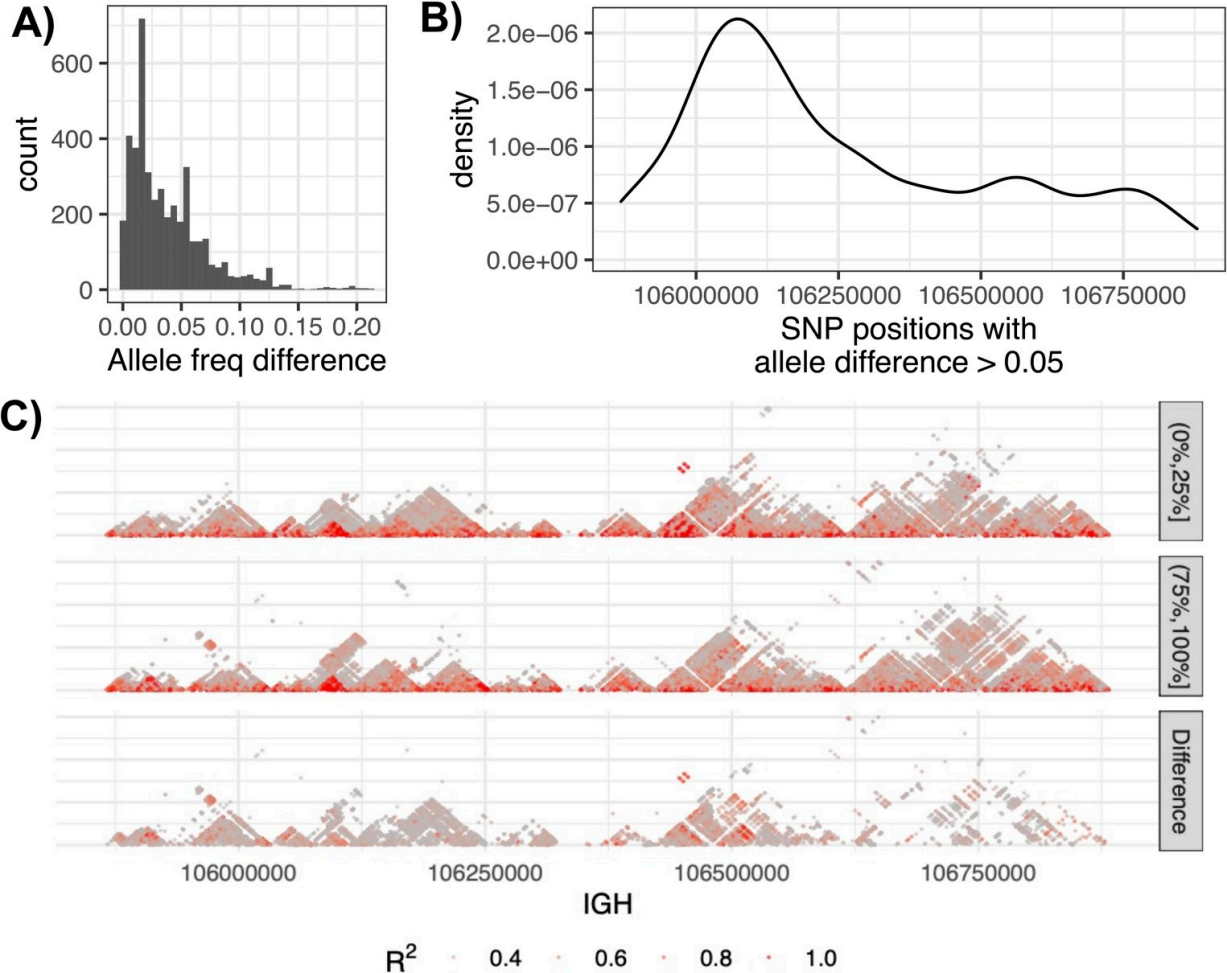
Differences in allele frequency and LD between individuals with low and high clonality. (A) The distribution of allele frequency differences at common SNPs (MAF >0.05) between individuals with low and high clonality, defined as samples in which 0 to 25% and 75% to 100% of sequencing data was derived from a single clone. (B) Position of common SNPs in IGH with allele frequency differences greater than 0.05. (C) LD for African individuals with low (“(0%,25%]”) and high clonality (“(75%,100%]”), and the difference in LD between both groups.

The previous section detailed the effects on genotypes due to V(D)J recombination. Given that genotypes are used to determine allele frequencies in a population, we set out to test if allele frequencies differed between samples that are more or less monoclonal. The allele frequencies of common SNPs (MAF > 0.05) were compared between samples within superpopulations with 0–25% (less monoclonal) and 75–100% (more monoclonal) of sequencing data derived from the dominant clone. Of the 4,354 SNPs analyzed, 1,258 (29%) had an allele frequency difference greater than 0.05 (Fig 5A). Since V(D)J recombination excises DNA 3’ of selected IGHV gene segments, we would expect to observe more genotyping errors caused by V(D)J related somatic deletions within the centromeric region of the IGHV locus. Consistent with this, we observed a greater number of SNPs exhibiting large differences in allele frequencies (>0.05) within the proximal (centromeric) region of the locus when comparing estimates generated from less monoclonal samples to more monoclonal samples (Fig 5B).

Genotypes are also used for the calculation of LD between SNPs. Given the demonstrated impact on allele frequency estimates, we reasoned that effects on genotype accuracy would also impact LD estimates. To assess this, we chose 76 samples from the African superpopulation representing extremes of clonality. Two groups of 38 samples each from the lower clonality group and the higher clonality group were selected. The LD *r*^2^ values across the locus were computed and compared between the two groups (Fig 5C), revealing different LD structure. We found that 11% (236,827) of the SNP pairs exhibited differences in LD (*r*^2^) greater than 0.1 (S4 Fig). The differences in allele frequencies and LD estimates observed here indicated that inaccurate genotypes resulting from impacts of V(D)J recombination also affect downstream analyses.

## Discussion

Previous studies have concluded that there are minimal differences between genotypes from matched LCL and non-LCL samples [10–13]. While true on a genome-wide scale, here we show that the impact on the IGH locus is more apparent due to V(D)J recombination. Using 1KGP samples (n = 2504) recently resequenced on the Illumina NovaSeq platform to 30x coverage using PCR-free 2x150 bp libraries, we evaluated different sequencing features affected by V(D)J recombination and SHM within the IGH locus. Specifically, we demonstrated that signatures of V(D)J recombination within LCL-derived DNA can be observed, including increased insert sizes of read mate-pairs, decreased read coverage over the IGHD and proximal IGHV gene segment regions, as well as direct evidence of somatically recombined IGHJ and IGHV gene segments. By assessing the frequency of specific IGHJ/IGHV recombination events within each sample, we were able to estimate the number of approximate B cell clones likely represented within a sample, and determine the proportion of sequencing data derived from each B cell clone, revealing variation in clonality across samples. Importantly, we were able to determine that V(D)J recombination can result in loss of DNA spanning large segments of the locus, with clear impacts on variant genotyping. The extent of these effects varied between samples based on the gene segments involved in the primary V(D)J recombination event, and the degree of monoclonality observed. Together these observations highlight critical limitations of using LCLs in combination with short read data to develop comprehensive reference resources for the IGH locus at the sample and population level.

It has previously been argued that the locus complexity of IGH has made it difficult to study using high-throughput approaches such as short read data and genotyping arrays [15–17]. This has impeded our ability to accurately characterize genetic diversity within IGH, and robustly test hypotheses about the functional role of IGH germline variation in disease risk and antibody-mediated immunity. The analyses we have conducted here indicate that the large-scale use of LCLs for establishing genetic reference panels in IGH may also present additional barriers to effectively interrogating IGH in genetic studies with downstream implications that need to be considered. For example, LCL-derived datasets such as the 1KGP have been critical for establishing population-genetic metrics across the genome, and have been used to augment GWAS and inform functional and population genetic studies. For example, consortia efforts such as gnomAD [23] have aggregated data from multiple sources, including LCL-derived data from the 1KGP, to power such studies. However, we have shown here that genotype, allele frequency, and LD estimates are incorrect for much of IGH due in part to impacts of V(D)J events in the data. This highlights a need to develop more specialized approaches for the continued use of LCLs for conducting genetic analyses in the IG loci. We argue that, at a minimum, the use of LCL-derived datasets could be improved by removing erroneous genotypes caused by V(D)J recombination induced deletions. As part of this study, we have released a BED file with the coordinates of V(D)J recombined induced deletions for each sample (S1 Table). It is possible that the development of genotyping pipelines that account for such data anomalies on a per-sample basis would lead to more accurate estimates of genotype and allele frequencies within IGH, with potential downstream implications for improving imputation approaches utilized by GWAS. Indeed, the use of long read sequencing to fully characterize IGH haplotypes from LCLs has been shown to resolve V(D)J derived deletions and provide accurate genotype calls [15]. Such analyses demonstrate that refined approaches can drastically improve the utility of using LCL repositories for IGH genetics.

Finally, while the focus of our study has been on the IGH locus, it is important to note that the observations we have made will also have relevance to the IGL and IGK loci as well. Moving forward, we argue that similar analyses should be conducted in IGL and IGK, and a more comprehensive effort should be undertaken to improve haplotype-based resources across the IG loci. Such efforts will be critical for effectively ensuring the inclusion of the IG loci in modern genetic studies.

## Supporting information

S1 Fig Prevalence of IGHV and IGHJ genes represented by dominant clones across samples. Heatmap showing the number of samples for which the dominant clone was represented by a given IGHJ-IGHV pair.
(TIF)

S2 Fig Fraction of samples per population exhibiting greater monoclonality. The fraction of samples per population in which either >50% (top) or >75% (bottom) of sequencing data was represented by the dominant clone.
(TIF)

S3 Fig Effect of V(D)J recombination and clonality on heterozygous SNP calling. (A) Scatterplot showing the number of heterozygous SNPs in the IGHV selected gene selected by V(D)J recombination relative to the percentage of sequencing data represented by the dominant clone in each individual of the 1KGP cohort. In each individual, the IGHV gene segment included in the analysis was based on the dominant clone. (B) Boxplot showing the comparison of the average number of heterozygous SNPs in IGHV genes selected for V(D)J recombination to all other genes; boxes represent calculations made across all samples, and each point represents a single individual.
(TIF)

S4 Fig Differences in LD for common SNPs in African individuals of varying monoclonality. Counts of SNP pairs at which differences in LD (R^2^) were observed between samples of low clonality (0–25%) and high clonality (75%-100%). Counts are shown for each range (category) of R^2^ differences, as indicated on the x axis.
(TIF)

S1 Table Coordinates of V(D)J recombined induced deletions. (TXT)
