# Supplementary figures and images for: Limitations of lymphoblastoid cell lines for establishing genetic reference datasets in the immunoglobulin loci
Source: PLoS One. 2021 Dec 13;16(12):e0261374. doi: 10.1371/journal.pone.0261374 (PMC8668129; doi:10.1371/journal.pone.0261374)

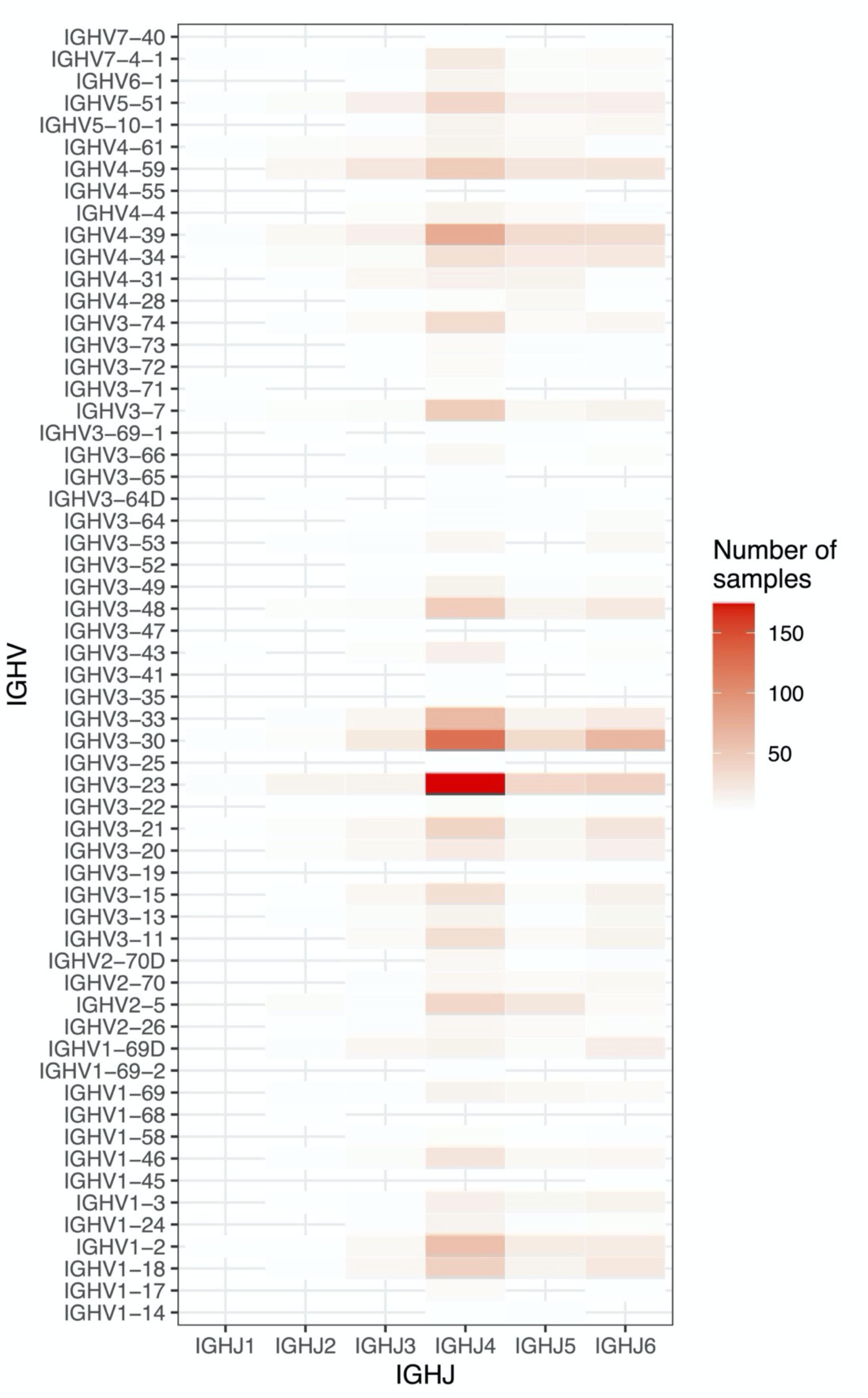

Supplement: S1 Fig — Heatmap showing the number of samples for which the dominant clone was represented by a given IGHJ-IGHV pair. (TIF) [file pone.0261374.s001.tif]

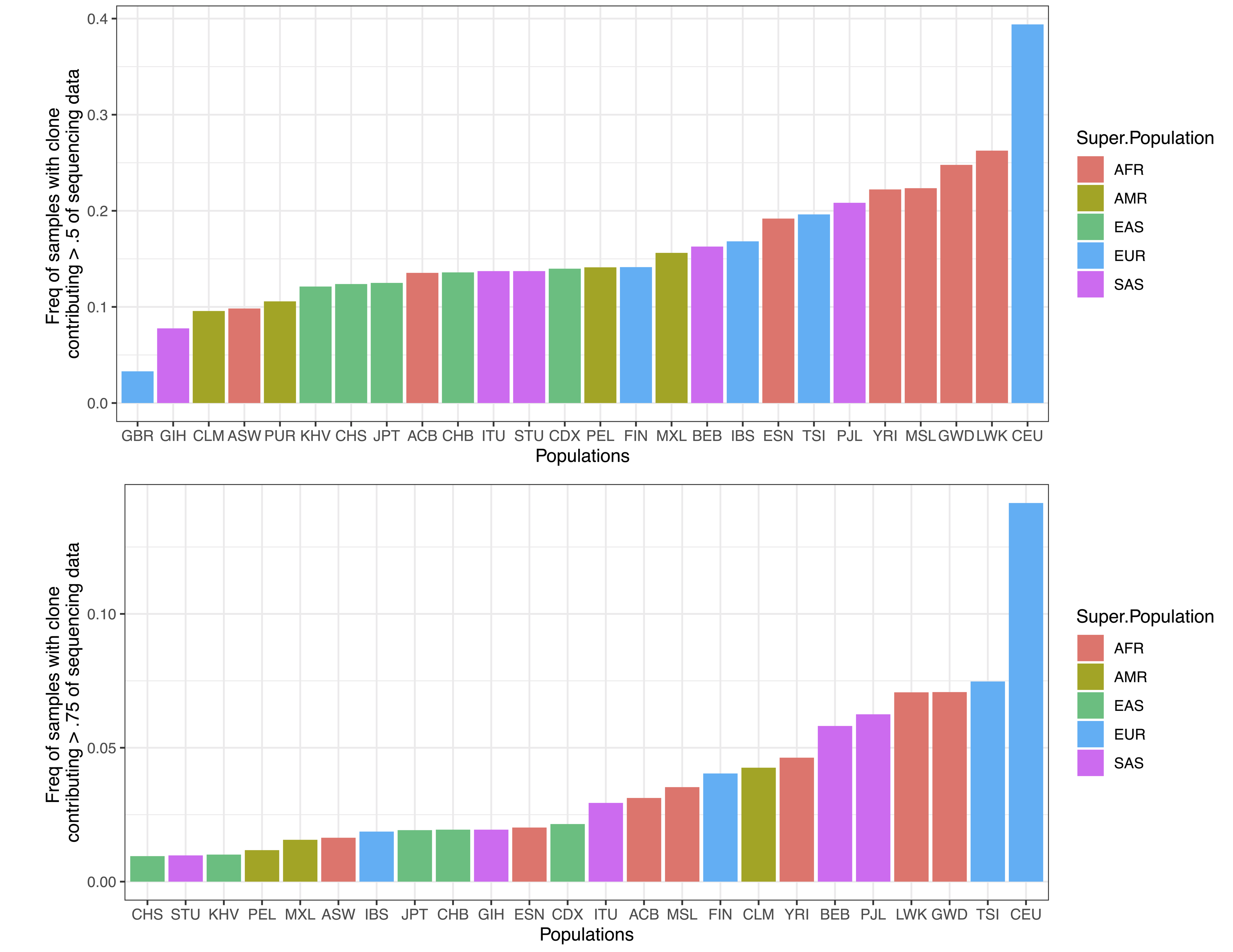

Supplement: S2 Fig — The fraction of samples per population in which either >50% (top) or >75% (bottom) of sequencing data was represented by the dominant clone. (TIF) [file pone.0261374.s002.tif]

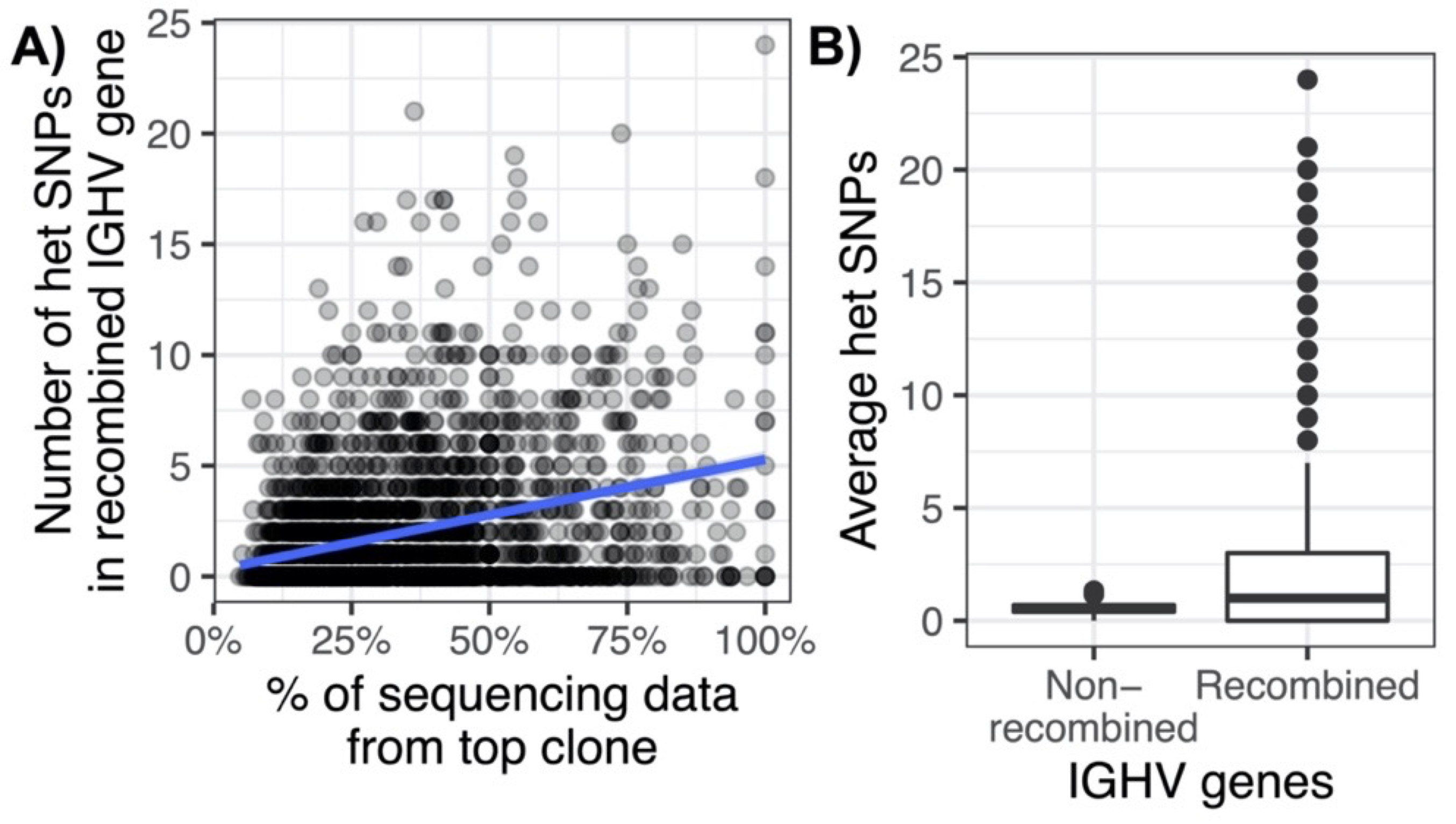

Supplement: S3 Fig — (A) Scatterplot showing the number of heterozygous SNPs in the IGHV selected gene selected by V(D)J recombination relative to the percentage of sequencing data represented by the dominant clone in each individual of the 1KGP cohort. In each individual, the IGHV gene segment included in the analysis was based on the dominant clone. (B) Boxplot showing the comparison of the average number of heterozygous SNPs in IGHV genes selected for V(D)J recombination to all other genes; boxes represent calculations made across all samples, and each point represents a single individual. (TIF) [file pone.0261374.s003.tif]

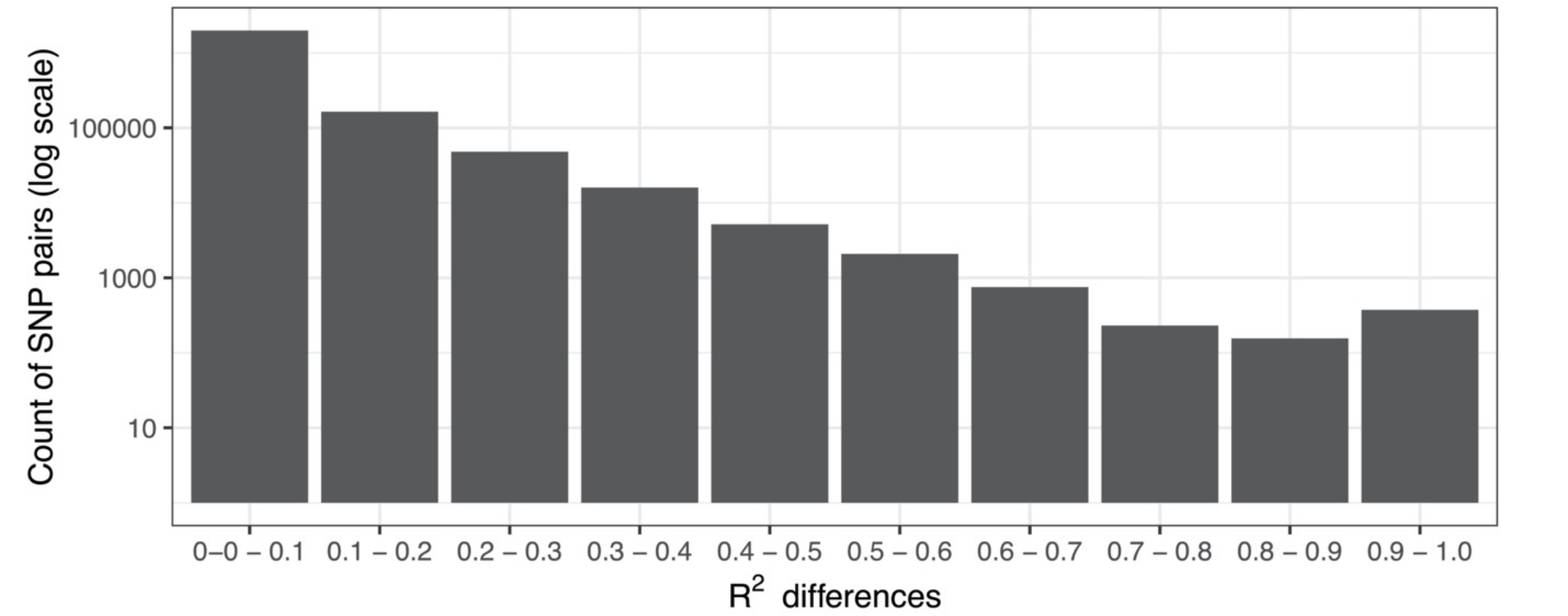

Supplement: S4 Fig — Counts of SNP pairs at which differences in LD (R2) were observed between samples of low clonality (0–25%) and high clonality (75%-100%). Counts are shown for each range (category) of R2 differences, as indicated on the x axis. (TIF) [file pone.0261374.s004.tif]
